# Supplementary material for: Essential Oil Headspace Volatiles Prevent Invasive Box Tree Moth (Cydalima perspectalis) Oviposition—Insights from Electrophysiology and Behaviour
Source: Insects. 2020 Jul 23;11(8):465. doi: 10.3390/insects11080465 (PMC7469176; doi:10.3390/insects11080465)
Supplement: Supplementary file 1 [file insects-11-00465-s001.pdf]

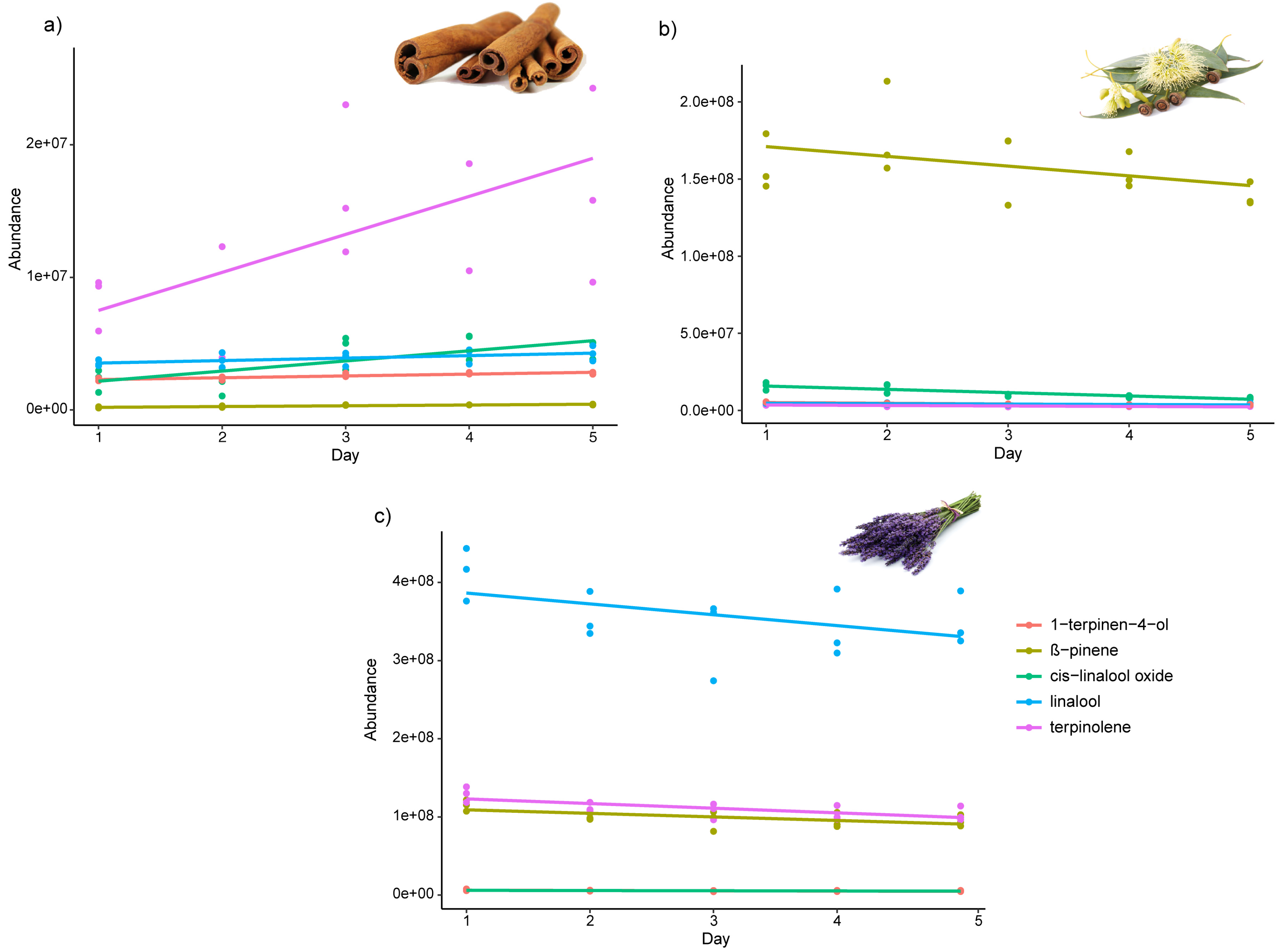

SFig.1. Evolution of the examined compounds over time in cinnamon (a), eucalyptus (b), and lavender (c). The fitted linear model shows trends for each of the compounds over time.

STable 1. Chemical composition of cinnamon, eucalyptus and lavender essential oils based on SPME headspace analysis

#	RT	RI NIST	RI Cal.	∆RI	CAS	Compound	Relative content (%)		
							Cinnamomum	Eucalyptus	Lavender
1	2,11	600	618	18	64-19-7	acetic acid	-	-	1,574
2	3,62	800	804	4	66-25-1	hexanal	0,119	-	-
3	4,33	855	856	1	6728-26-3	trans-2-Hexenal	0,017	-	-
4	4,38	855	860	5	928-96-1	*cis*-3-Hexen-1-ol	-	-	0,028
5	4,49	868	868	0	100-41-4	ethylbenzene	0,030	-	-
6	4,54	867	871	4	111-27-3	1-Hexanol	-	-	0,076
7	5,01	898	905	7	100-42-5	styrene	1,231	-	-
8	5,59	948	943	5	80-56-8	*α*-Pinene	1,642	8,310	4,916
9	5,83	952	959	7	79-92-5	camphene	0,738	0,168	0,443
10	5,98	966	969	3	100-52-7	benzaldehyde	8,226	-	-
11	6,20	977	983	6	3387-41-5	sabinene	-	-	0,135
12	6,34	975	993	18	106-68-3	3-Octanone	-	-	0,632
13	6,27	980	988	8	127-91-3	*β*-Pinene	0,200	1,541	6,704
14	6,30	988	990	2	110-93-0	6-methyl-5-hepten-2-one	0,138	-	-
15	6,42	992	997	5	123-35-3	*β*-Myrcene	0,462	-	-
16	6,55	1003	1006	3	271-89-6	benzofuran	0,062	-	-
17	6,63	1006	1011	5	499-97-8	*p*-Mentha-1(7),8-diene	0,016	-	-
18	6,68	1006	1014	8	99-83-2	*α*-Phellandrene	7,562	0,196	-
19	6,73	1007	1018	11	13466-78-9	3-Carene	0,011	-	-
20	6,77	1014	1020	6	142-92-7	hexylacetate	-	-	1,389
21	6,83	1017	1024	7	99-86-5	*α*-Terpinene	0,012	-	-
22	6,98	1022	1034	12	99-87-6	*p*-Cymene	10,522	1,024	0,025
23	7,03	1027	1037	10	138-86-3	limonene	0,073	1,087	3,439
24	7,08	1033	1040	7	470-82-6	eucalyptol	0,194	76,964	12,618
25	7,18	1031	1047	16	535-77-3	*m*-Cymene	0,009	-	-
26	7,33	1037	1056	19	3338-55-4	*cis-β*-Ocimene	1,495	-	3,318
27	7,42	1057	1062	5	2548-87-0	*trans*-2-Octenal	0,005	-	-

28	7,48	1060	1066	6	99-85-4	*γ* -Terpinene	0,153	-	0,127
29	7,64	1060	1076	16	15537-55-0	*cis* -Sabinene hydrate (4-Thuja	-	-	0,009
30	7,60	1066	1074	8	98-86-2	acetophenone	0,295	0,158	-
31	7,74	1072	1083	11	34995-77-2	*trans* -Linalool oxide (furanoid)	-	-	0,370
32	7,69	1078	1080	2	5989-33-3	*cis* -Linalool oxide	0,048	0,111	0,672
33	7,95	1090	1096	6	586-62-9	terpinolene	0,180	0,567	0,344
34	8,07	1100	1104	4	78-70-6	linalool	0,802	0,053	24,072
35	8,13	1102	1108	6	124-19-6	nonanal	0,107	-	-
36	8,21	1103	1114	11	659-70-1	isoamyl valerianate	-	0,665	-
37	8,25	1095	1116	21	nd	*α* -Pinene epoxide	-	0,045	-
38	8,32	1114	1121	7	60-12-8	phenylethyl Alcohol	1,074	-	-
40	8,47	1123	1131	8	4864-61-3	3-Octylacetate	-	-	0,087
41	8,58	1144	1138	6	7216-56-0	(E,Z)-2,6-dimethyl-2,4,6-Octatr	0,006	-	0,755
42	8,61	1150	1140	10	91819-58-8	*α* -Campholenal	-	0,774	-
43	8,66	1136	1143	7	586-82-3	3-Terpinen-1-ol	0,184	-	-
44	8,71	1145	1147	2	13837-75-7	*cis* -Limonene-oxide	-	1,280	-
45	8,77	1143	1151	8	4959-35-7	*trans* -Limonene-oxide	-	0,616	-
46	8,78	1132	1151	19	57396-75-5	3,4-Dimethyl-2,4,6-octatriene	0,034	-	0,072
47	8,82	1143	1154	11	138-87-4	*β* -Terpineol	0,077	-	-
48	8,84	1140	1155	15	547-61-5	trans-Pinocarveol	-	0,409	-
49	8,88	1144	1158	14	473-67-6	verbenol	-	0,145	-
50	8,94	1144	1162	18	464-49-3	(+)-Camphor	0,262	-	11,701
51	9,07	1160	1170	10	104-53-0	benzenepropanal	2,068	-	-
52	9,14	1159	1175	16	547-60-4	pinocamphone	-	0,159	-
53	9,16	1165	1176	11	498-16-8	(-)-Lavandulol	-	-	0,058
54	9,17	1161	1177	16	30460-92-5	pinocarvone	-	0,423	-
55	9,22	1169	1180	11	507-70-0	borneol	0,496	-	2,145
56	9,40	1177	1192	15	562-74-3	1-Terpinen-4-ol	0,978	0,065	0,387
57	9,49	1186	1198	12	500-02-7	crypton	-	0,309	0,034
58	9,48	1189	1197	8	98-55-5	*α* -Terpineol	0,554	-	-

59	9,44	1190	1194	4	nd	*n*-Hexylbutyrate	-	-	0,937
61	9,52	1200	1200	0	112-40-3	dodecane	0,044	-	-
62	9,54	1189	1201	12	98-55-5	α-Terpineol	-	1,467	0,168
63	9,60	1194	1205	11	515-00-4	myrtenol	-	0,377	-
64	9,63	1205	1208	3	112-31-2	decanal	0,133	-	-
65	9,64	1194	1208	14	564-94-3	myrtenal	-	0,290	-
66	9,76	1200	1217	17	5948-04-09	*trans*-Dihydrocarvone	-	0,028	-
67	9,84	1206	1223	17	80-57-9	verbenone	-	0,396	-
68	9,92	1214	1229	15	1197-07-5	*trans*-Carveol	-	0,536	0,007
69	9,95	1229	1230	1	18679-48-6	1,8-epoxy-p-Menthan-2-ol	-	0,028	-
70	10,05	1239	1238	**1**	106-25-2	nerol	-	-	0,051
71	10,09	1226	1241	15	1197-06-4	*cis*-Carveol	-	0,041	-
72	10,11	1222	1242	20	125-12-2	isobornylacetate	-	-	0,115
73	10,15	1241	1245	4	10632-13-0	hexyl isovalerate	-	-	0,061
74	10,24	1239	1252	13	122-03-2	cumic aldehyde	0,052	0,083	-
75	10,25	1258	1252	6	135-02-4	2-Anisaldehyde	1,876	-	-
76	10,31	1254	1256	2	99-49-0	carvone	-	0,661	-
77	10,32	1244	1257	13	499-71-8	carvotanacetone	-	0,052	-
78	10,42	1256	1264	8	103-45-7	2-Phenethyl acetate	0,383	-	-
79	10,47	1257	1268	11	115-95-7	linalyl acetate	-	-	11,760
81	10,65	1260	1280	20	5392-40-5	citral	-	-	0,007
82	10,80	1283	1291	8	14371-10-9	trans-Cinnamaldehyde	49,818	0,040	0,005
83	10,85	1289	1295	6	104-46-1	anethole	0,523	-	-
84	10,86	1292	1295	3	20777-39-3	lavandulyl acetate	-	-	0,234
85	10,89	1289	1298	9	76-49-3	bornyl acetate	-	-	0,036
86	10,91	1285	1299	14	5655-61-8	(-)-Bornyl acetate	0,133	-	-
87	10,92	1295	1300	5	536-59-4	*p*-Mentha-1,8-dien-7-ol	-	0,017	-
88	10,92	1300	1300	0	629-50-5	tridecane	-	0,030	-
89	11,03	1300	1308	8	105-86-2	geranylformate	-	-	0,005
90	11,53	1342	1346	4	1946-00-5	trans-8-p-Menthadien-1,2-diol	-	0,018	-

91	11,66	1355	1356	1	64142-78-5	8-hydroxylinalool	-	-	0,007
92	11,72	1350	1361	11	80-26-2	*α*-Terpinyl acetate	-	0,028	0,107
93	11,77	1351	1364	13	17699-14-8	*α*-Cubebene	0,015	-	0,003
94	11,84	1362	1370	8	141-12-8	neryl acetate	-	0,002	1,852
95	11,80	1359	1367	8	97-53-0	eugenol	0,013	-	-
96	11,84	1402	1370	32	475-20-7	longifolene	0,010	-	-
97	12,03	1370	1384	14	22469-52-9	cyclosativene	0,256	-	-
98	12,08	1372	1388	16	14912-44-8	ylangene	0,145	0,034	-
99	12,11	1386	1390	4	105-87-3	geranylacetate	-	-	2,910
101	12,15	1376	1393	17	3856-25-5	*α*-Copaene	2,013	-	0,014
102	12,28	1384	1403	19	5208-59-3	*β*-Bourbonene	0,072	-	0,012
103	12,30	1391	1405	14	515-13-9	*β*-Elemene	0,049	0,005	-
104	12,43	1396	1415	19	3650-28-0	(+)-Sativene	0,037	-	-
105	12,47	1428	1418	10	13474-59-4	*trans-α* -Bergamotene	-	-	0,009
106	12,59	1408	1428	20	469-61-4	*α*-cedrene	0,071	-	-
107	12,71	1420	1437	17	512-61-8	*α*-santalene	-	-	0,026
108	12,74	1444	1440	4	87-44-5	*β*-Caryophyllene	0,183	0,018	1,626
109	12,85	1476	1448	28	495-60-3	*α*-Zingiberene	0,145	-	-
110	12,92	1452	1454	2	103-54-8	cinnamyl acetate	1,042	-	-
111	12,94	1440	1456	16	17334-55-3	*β*-Gurjunene	-	0,019	-
112	12,99	1440	1459	19	489-39-4	aromadendrene	0,106	0,391	-
113	13,06	1443	1465	22	28973-97-9	*cis-β* -farnesane	-	-	0,032
114	13,20	1456	1476	20	6753-98-6	*α*-Humulene	-	-	0,014
115	13,27	1467	1481	14	25246-27-9	alloaromadendrene	0,141	0,033	-
116	13,42	1476	1493	17	30021-74-0	*γ*-Muurulene	0,252	-	-
117	13,59	1495	1507	12	88-84-6	*β*-Guaiene	0,020	-	-
118	13,61	1490	1509	19	17066-67-0	eudesma-4(14),11-diene	-	0,005	-
119	13,64	1507	1512	5	39029-41-9	*γ*-Cadinene	0,008	-	-
120	13,67	nd	1514	-	2306-78-7	nerolidyl acetate	-	-	0,004
122	13,69	1497	1516	19	31983-22-9	*α*-Muurolene	0,103	-	

123	13,70	1500	1517	17	nd	linalyl isovalerate	-	-	0,001
124	13,74	1508	1520	12	495-61-4	*β*-Bisabolene	0,075	-	
125	13,77	1533	1522	11	128-37-0	butylated hydroxytoluene	-	0,045	-
126	13,92	1534	1535	1	39029-41-9	*γ*-Cadinene	-	-	0,011
127	13,98	1523	1539	16	483-76-1	*δ*-Cadinene	0,126	-	
128	14,02	1543	1543	0	483-77-2	calamenene	-	0,001	-
129	14,03	1536	1544	8	1963-36-6	4-Methoxycinnamaldehyde	0,469		-
130	14,70	1600	1600	0	544-76-3	hexadecane	-	0,003	-
131	14,80	1613	1609	4	1139-30-6	caryophyllene oxide	-	-	0,006
132	14,81	1584	1609	25	51371-47-2	globulol	-	0,002	
133	15,22	1635	1645	10	14035-34-8	2,6-Bis(1,1-dimethylethyl)-4-(1-oxopropyl)phenol	-	0,003	-
134	15,28	n.a.	1650	-	121917-12-2	Endo-2,3-di(methoxycarbonyl)-benzo[e]bicyclo[2.2.2]octane	-	0,004	-
135	15,81	1677	1696	19	483-78-3	cadalene	-	0,002	-
136	15,83	1700	1698	2	629-78-7	heptadecane	-	0,004	-
	**Total**						**98,392**	**99,729**	**96,152**
